# Efficacy of 5-ALA Photodynamic Therapy in Dysplastic Oral Leukoplakia: Systematic Review and Meta-Analysis

**DOI:** 10.3390/pharmaceutics18020254

**Published:** 2026-02-18

**Authors:** Magdalena Sulewska, Patryk Wiśniewski, Monika Stępniewska, Zuzanna Poloczek, Dawid Chodziński, Piotr Melion, Maksymilian Pawluczuk, Aleksandra Pietruska, Małgorzata Pietruska

**Affiliations:** 1 Department of Periodontal and Oral Mucosa Diseases, Medical University of Białystok, ul. Waszyngtona 13, 15-269 Bialystok, Poland; patryk.wisniewski@umb.edu.pl (P.W.); malgorzata.pietruska@umb.edu.pl (M.P.); 2 Students’ Research Group on Oral Mucosal Diseases, Department of Periodontal and Oral Mucosa Diseases, Medical University of Białystok, ul. Waszyngtona 13, 15-269 Bialystok, Poland; 45029@student.umb.edu.pl (M.S.); 45025@student.umb.edu.pl (Z.P.); 44989@student.umb.edu.pl (D.C.); 45017@student.umb.edu.pl (P.M.); 45020@student.umb.edu.pl (M.P.); perio@umb.edu.pl (A.P.)

**Keywords:** oral leukoplakia, epithelial dysplasia, photodynamic therapy, 5-aminolevulinic acid, meta-analysis, recurrence, malignant transformation

## Abstract

**Background**: Oral leukoplakia (OL) with oral epithelial dysplasia (OED) carries an increased risk of malignant transformation and typically requires active management and long-term surveillance. Surgical excision remains the gold standard, yet recurrence is common and morbidity may be substantial, particularly in extensive or multifocal disease. Photodynamic therapy using 5-aminolevulinic acid (ALA-PDT) has emerged as a minimally invasive alternative. However, its effectiveness in dysplastic OL has not been quantified systematically. **Methods**: A systematic review and meta-analysis were conducted according to a prospectively registered protocol (PROSPERO: CRD420251249586) and reported in line with PRISMA 2020. PubMed, Scopus, and Web of Science were searched from inception to 15 December 2025 for clinical studies evaluating ALA-PDT as primary treatment for OL with histopathologically confirmed OED. Single-arm prospective or retrospective studies reporting clinical response were eligible. Risk of bias was assessed using the Joanna Briggs Institute Critical Appraisal Checklist for Case Series. Pooled overall response rate (ORR) and complete response (CR) were estimated as proportions using random-effects models. Recurrence and malignant transformation were summarized as incidence rates per 100 person-years. **Results**: Six single-arm clinical studies including 109 patients with dysplastic oral leukoplakia treated with 5-ALA-mediated photodynamic therapy were eligible for quantitative synthesis. The pooled overall response rate was 0.85 (95% CI 0.74–0.93), whereas the pooled complete response rate reached 0.34 (95% CI 0.18–0.53), with moderate to substantial heterogeneity. Recurrence and malignant transformation outcomes were limited and analyzed descriptively, suggesting low but persistent long-term risk. The overall certainty of the evidence was rated as very low according to GRADE. **Conclusions**: 5-ALA-mediated photodynamic therapy appears to be an effective and minimally invasive treatment option for oral leukoplakia with epithelial dysplasia; however, the very low certainty of evidence, lack of standardized protocols, and persistent risk of recurrence and malignant transformation highlight the need for well-designed controlled studies and long-term clinical surveillance.

## 1. Introduction

Oral leukoplakia (OL) is defined as a white patch or plaque of the oral mucosa that cannot be scraped off and cannot be classified as any other known disease entity [1,2]. The etiology of OL is multifactorial and primarily includes tobacco smoking and alcohol consumption, as well as chronic local irritation, inadequate oral hygiene, Candida spp. and HPV infections, nutritional deficiencies, and certain hereditary disorders [3,4,5,6]. Owing to its potential for malignant transformation, OL requires particular diagnostic and therapeutic vigilance.

Histopathological examination of a biopsy specimen remains the diagnostic gold standard for OL. Despite the development of non-invasive adjunctive techniques, including methods supported by artificial intelligence, histopathological assessment still determines the diagnosis and guides subsequent clinical management [3,4,7]. The clinical relevance of OL is further underscored by the fact that it is the most common oral potentially malignant disorder (OPMD), a heterogeneous group of mucosal conditions that may progress to oral squamous cell carcinoma (OSCC). According to data from recent meta-analyses, the cumulative risk of malignant transformation across OPMDs ranges from 3% to 50%, whereas for OL it is estimated at 17–35% [8,9].

One of the key prognostic factors for malignant transformation in OL is oral epithelial dysplasia (OED) [10]. OED reflects histopathological architectural and cytological abnormalities associated with increased oncogenic risk. However, not every dysplastic lesion progresses to malignancy and some may undergo spontaneous regression [11,12,13,14]. The severity of dysplasia (mild, moderate and severe) is graded using the three-tier WHO classification, with the 2017 revision incorporating carcinoma in situ into the category of severe dysplasia [15,16]. The risk of malignant transformation increases with dysplasia grade and has been reported as 5.23%, 12.57%, and 24.98% for mild, moderate, and severe dysplasia, respectively [15,17].

Management of dysplastic OL includes both surgical and non-surgical approaches. Chemoprevention (e.g., vitamin A, retinoids and carotenoids) shows variable efficacy and notable limitations [17,18,19]. Surgical treatment (excision, cryotherapy, CO_2_ laser ablation, and electrocautery) is widely used. However, recurrence occurs in 10–35% of patients and the invasiveness of these procedures may limit their applicability, particularly in extensive and multifocal lesions [17,18,20,21,22,23,24,25].

Therefore, there is growing interest in photodynamic therapy (PDT) as a minimally invasive, well-tolerated modality with potential utility in multifocal disease [21,24,26]. PDT relies on the interaction of a photosensitizer, light of an appropriate wavelength, and tissue oxygen, resulting in the generation of reactive oxygen species and selective destruction of diseased cells [21,26,27]. One of the photosensitizers used in OL is 5-aminolevulinic acid (5-ALA), an endogenous precursor of protoporphyrin IX (PpIX), which preferentially accumulates in dysplastic and proliferating cells due to reduced ferrochelatase activity and impaired conversion to heme [21,28]. Despite an increasing number of clinical reports, the effectiveness of ALA-PDT in dysplastic OL remains insufficiently established, partly due to the limited number of studies, heterogeneity of treatment protocols, and lack of standardization [26,29,30]. In addition, previously published systematic reviews and meta-analyses have generally evaluated photodynamic therapy across multiple photosensitizers and without consistent stratification according to histopathologically confirmed epithelial dysplasia, which may contribute to clinical and methodological heterogeneity and limit treatment-specific interpretation of outcomes [26,31,32].

The aim of this study is to conduct a systematic review and meta-analysis to assess the efficacy of ALA-PDT in the treatment of oral leukoplakia with histopathologically confirmed epithelial dysplasia.

## 2. Materials and Methods

### 2.1. Search Strategy, Eligibility Criteria and Data Extraction

The review protocol was prospectively registered in PROSPERO (registration number: CRD420251249586) and the methods followed the recommendations of the Cochrane Handbook and the PRISMA 2020 statement [33,34]. This systematic review and meta-analysis addressed the clinical effectiveness of ALA-PDT for the management of OL with histopathologically confirmed epithelial dysplasia.

A comprehensive electronic search was carried out in three databases: PubMed, Scopus and Web of Science. Google Scholar was not used as a primary source because of limited transparency of its search algorithm, inconsistent indexing practices and the inclusion of non-peer-reviewed material. Embase and the Cochrane Library were not included due to the lack of institutional access at the time of the search; however, the combined use of PubMed, Scopus and Web of Science provides broad coverage of biomedical literature, including numerous European journals and conference-indexed records. Gray literature sources (e.g., clinical trial registries or conference abstracts) were not systematically searched because many such records lack complete quantitative outcome data and are not peer-reviewed, which limits their suitability for meta-analytic synthesis. Detailed search strategies for each database are presented below.

1. For PubMed: ((leukoplakia[Title/Abstract] OR “oral leukoplakia”[Title/Abstract] OR leucoplakia[Title/Abstract] OR leucoplasia[Title/Abstract]) AND (PDT[Title/Abstract] OR photodynamic[Title/Abstract] OR “photodynamic therapy”[Title/Abstract] OR “photodynamic treatment”[Title/Abstract] OR ALA[Title/Abstract] OR “5-ALA”[Title/Abstract] OR “ALA-PDT”[Title/Abstract]))
2. For Scopus: TITLE-ABS-KEY((leukoplakia OR “oral leukoplakia” OR leucoplakia OR leucoplasia) AND (PDT OR photodynam* OR “photodynamic therap*” OR “photodynamic treatment” OR ALA OR “5-ALA” OR “ALA-PDT”))
3. For Web of Science: TS = ((leukoplakia OR “oral leukoplakia” OR leucoplakia OR leucoplasia) AND (PDT OR photodynamic OR “photodynamic therapy” OR “photodynamic treatment” OR ALA OR “5-ALA” OR “ALA-PDT”))

The first search was conducted on 10 December 2025, and the last on 15 December 2025. We aimed to identify interventional and observational single-arm studies evaluating photodynamic therapy as the primary treatment for oral leukoplakia with histopathologically confirmed epithelial dysplasia. No restrictions on year of publication were applied, but the search was limited to articles published in English and involving human participants. We excluded studies in which PDT was used solely for diagnostic purposes, studies not providing separate data for dysplastic oral leukoplakia, non-interventional designs without analyzable treatment outcomes, case reports, reviews, conference abstracts, experimental animal or in vitro studies and articles with insufficient quantitative data for extraction or inclusion in the meta-analysis.

Titles and abstracts were independently screened by two reviewers (Z.P. and M.St.), followed by full-text assessment to determine eligibility based on predefined PICOS-based inclusion and exclusion criteria for ALA-PDT in dysplastic oral leukoplakia (Table 1). Any disagreements were resolved by discussion and, when consensus could not be reached, by consultation with a third reviewer (P.W.). Potential duplicate records were identified and removed using Zotero 7.0.30 reference management software by both reviewers (Z.P. and M.St.).

For studies that did not provide sufficient quantitative information, we attempted to contact the corresponding authors to obtain the original data. If no response was received or the necessary data could not be retrieved, the study was excluded from the quantitative synthesis (meta-analysis), but could still be retained in the qualitative review if it met the eligibility criteria.

From each eligible study, we extracted:

1. Article-level information—first author, year of publication, country, journal, study design, clinical setting, sample size, follow-up duration.
2. Participant and lesion characteristics—number of patients and/or lesions, age, sex, lesion location, lesion size or extent, dysplasia grade, and relevant risk factors.
3. Intervention details—photodynamic protocol restricted to ALA-based PDT: photosensitiser formulation and concentration, route and time of application, light source and wavelength, fluence, power density, number and frequency of treatment sessions, and any concomitant therapies.
4. Outcome data—numbers or proportions of lesions with complete response, partial response and no response; data on recurrence after initial response, malignant transformation, as well as definitions of clinical response, recurrence and malignant transformation used in each study.

### 2.2. Quality Assessment

The methodological quality and risk of bias of the included single-arm ALA-PDT studies were assessed using the Joanna Briggs Institute (JBI) Critical Appraisal Checklist for Case Series, which evaluates ten domains related to eligibility criteria, consistency and validity of diagnosis, completeness of inclusion and follow-up, reporting of demographic and clinical details, clarity of outcome reporting, adequacy of follow-up, and appropriateness of the statistical analysis [35]. Each item was judged as “yes”, “no”, “unclear” or “not applicable”. For the purposes of this review, studies with at least eight items rated “yes” and no key domain rated “no” were classified as having low risk of bias, those with five to seven “yes” ratings or a single “no” in a key domain as a moderate risk, and those with fewer than five “yes” ratings or two or more “no” ratings in key domains as a high risk of bias.

### 2.3. Statistical Analysis

Quantitative synthesis was performed when at least two independent studies reported the same outcome. For overall response rate (ORR) and complete response (CR), effect estimates were calculated as study-level proportions (events/total) with 95% confidence intervals. Pooled effect sizes were computed using a random-effects meta-analysis, given the anticipated clinical and methodological diversity across studies (e.g., variability in PDT protocols, follow-up duration, and patient or lesion characteristics). Between-study variance (τ^2^) was estimated using the restricted maximum likelihood (REML) method. Proportion data were stabilized using the Freeman-Tukey double arcsine transformation. Subgroup meta-analyses were conducted according to epithelial dysplasia grade (mild, moderate, severe) where data were available. Statistical heterogeneity was quantified using the I^2^ statistic and τ^2^, and pooled results were reported with 95% confidence intervals.

Recurrence outcomes were inconsistently reported and associated with heterogeneous follow-up durations; therefore, recurrence was summarized using incidence rates (IRs), calculated as the number of recurrence events divided by the estimated person-time at risk (N × mean follow-up in years when person-time was not directly reported), and expressed per 100 person-years with 95% Poisson confidence intervals. A crude overall recurrence incidence rate was additionally estimated by aggregating events and person-time across studies reporting recurrence. Malignant transformation outcomes were analyzed descriptively; when reported in a single study, an incidence rate was calculated analogously, whereas meta-analysis was not performed due to the insufficient number of studies.

Sensitivity analyses were conducted using a leave-one-out approach within the random-effects framework to evaluate the influence of individual studies on pooled estimates and heterogeneity. Assessment of small-study effects or publication bias (e.g., funnel plot asymmetry) was not performed because the number of studies per meta-analysis was below the recommended threshold (k < 10) [36]. All statistical analyses were performed using Stata/BE version 19.5 (StataCorp, College Station, TX, USA).

## 3. Results

### 3.1. Literature Searches

**Figure 1:**
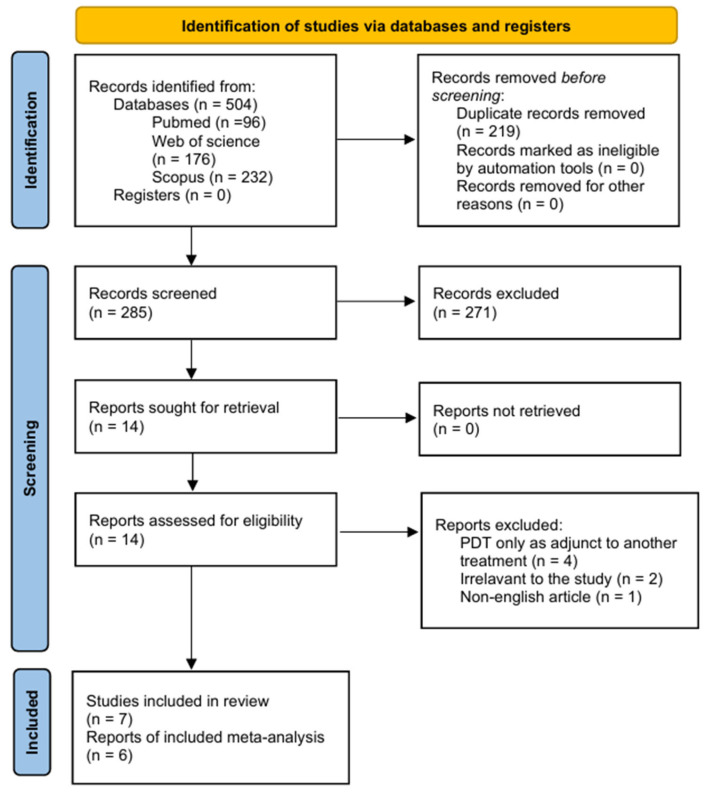
PRISMA flow chart. (Accessed on 15 December 2025).

The study selection process is summarized in the PRISMA flow diagram (Figure 1). The electronic search of PubMed, Scopus and Web of Science identified a total of 504 records up to 15 December 2025 (96 from PubMed, 232 from Scopus and 176 from Web of Science). After the removal of 219 duplicate records, 285 unique records remained for title and abstract screening. Of these, 271 were excluded as clearly irrelevant (e.g., studies not involving oral leukoplakia with dysplasia, irrelevant protocol of treatment, animal or in vitro studies and reviews).

Fourteen full-text articles were sought for retrieval. All of the reports were retrieved. Fourteen full-text articles were assessed for eligibility, and seven were excluded because they did not meet the predefined PICO criteria. Ultimately, seven studies fulfilled the inclusion criteria and were included in the qualitative synthesis [29,37,38,39,40,41,42]. However, because one study did not report sufficiently detailed quantitative data, only six studies could be included in the meta-analysis [29,37,38,39,40,41].

### 3.2. Study Characteristics

A total of six eligible studies are summarized in detail in Table 2. Altogether, these single-arm studies included 109 patients with dysplastic OL. For the quantitative synthesis, we considered four types of outcomes: complete response, overall response, recurrence, and malignant transformation. All data on the ALA-PDT protocol of each study are presented in Table 3. Notable differences were observed in the drug-light interval (ranging from 1.5 to 2 h), as well as total session duration (the shortest being 300 s, while most lasted 1000 s), fluence rate (ranging from 100 to 500 mW/cm^2^), light exposure dose and general treatment schedule (ranging from a single session to biweekly applications). A consistent 20% concentration of ALA was used across all studies; however, details regarding its formulation (e.g., cream, gel) were provided in merely half of the reports.

All included studies used a single-arm design, and patients were diagnosed with OL with epithelial dysplasia on clinical and histopathological grounds.

**Figure 2:**
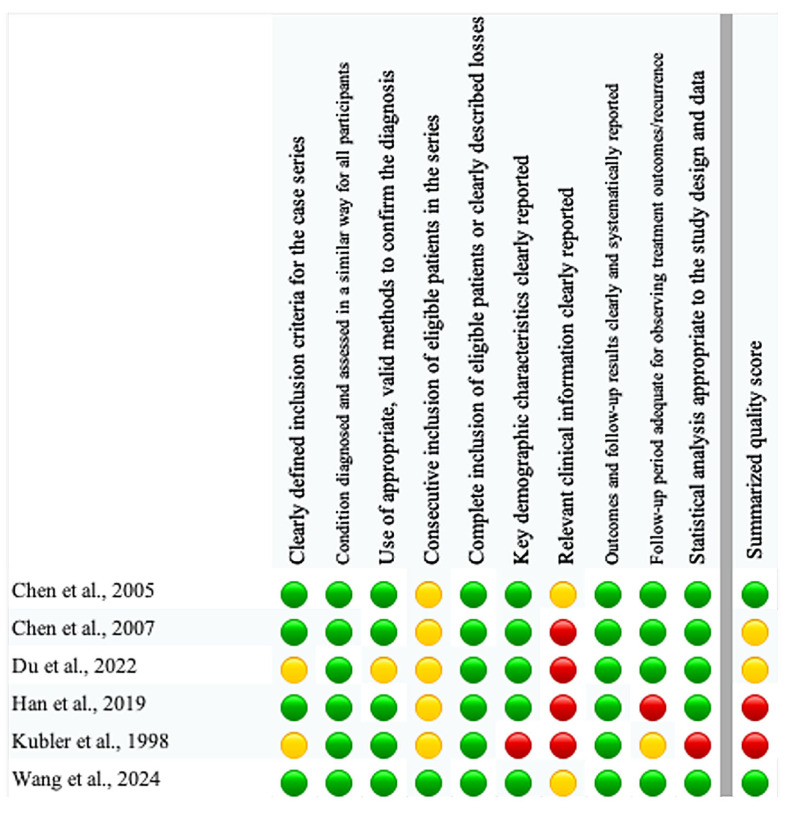
Risk of bias assessment [29,37,38,39,40,41].

According to our predefined thresholds, two studies were judged to have a low risk of bias, two studies were considered to have a moderate risk of bias, and two studies were classified as high risk of bias (Figure 2).

### 3.3. Overall Response Rate (ORR)

ORR after photodynamic therapy was synthesized using a random-effects model. The pooled ORR in the overall analysis was 0.85 (95% CI 0.74–0.93), with low-to-moderate between-study heterogeneity (I^2^ = 33.4%).

**Figure 3:**
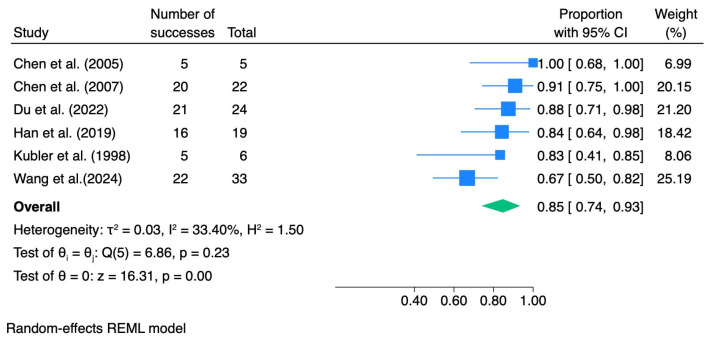
Forest plot—ORR all dysplasia. Blue squares represent individual study effect estimates with 95% confidence intervals; the green diamond represents the pooled overall effect estimate with its 95% confidence interval [29,37,38,39,40,41].

**Figure 4:**
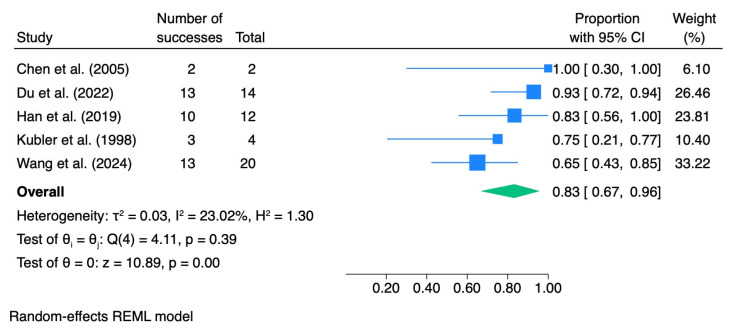
Forest plot—ORR mild dysplasia. Blue squares represent individual study effect estimates with 95% confidence intervals; the green diamond represents the pooled overall effect estimate with its 95% confidence interval [29,37,39,40,41].

**Figure 5:**
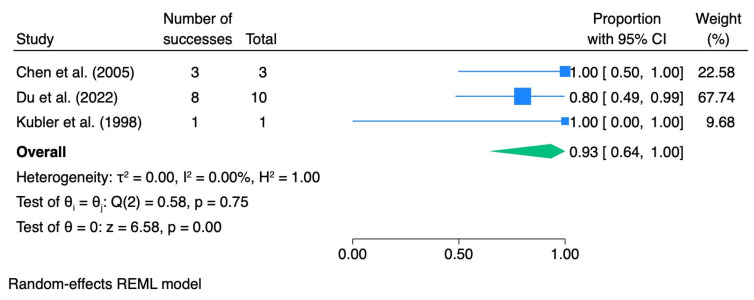
Forest plot—ORR moderate dysplasia. Blue squares represent individual study effect estimates with 95% confidence intervals; the green diamond represents the pooled overall effect estimate with its 95% confidence interval [37,39,41].

**Figure 6:**
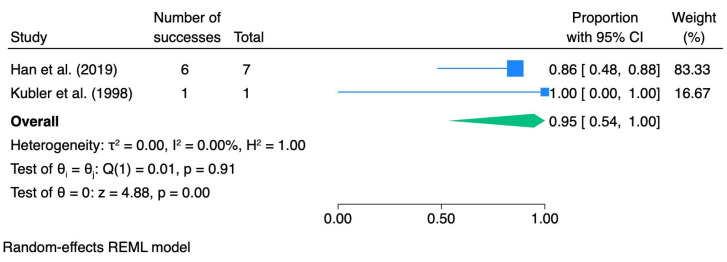
Forest plot—ORR severe dysplasia. Blue squares represent individual study effect estimates with 95% confidence intervals; the green diamond represents the pooled overall effect estimate with its 95% confidence interval [40,41].

In subgroup analyses stratified by the grade of epithelial dysplasia, the pooled ORR was 0.83 (95% CI 0.67–0.96; I^2^ = 23.02%) for mild dysplasia, 0.93 (95% CI 0.64–1.00; I^2^ = 0.0%) for moderate dysplasia, and 0.95 (95% CI 0.54–1.00; I^2^ = 0.0%) for severe dysplasia. Table 4 summarizes the meta-analytic results for dysplasia grade, including pooled effect sizes with 95% confidence intervals and heterogeneity statistics. The corresponding forest plots are presented in Figure 3, Figure 4, Figure 5 and Figure 6.

For subgroup-specific analyses, studies that reported aggregated outcomes across multiple dysplasia grades were excluded to avoid double counting and misclassification. Chen et al. (2007) was excluded from the moderate dysplasia subgroup because the reported ORR combined mild and moderate lesions, whereas Wang et al. (2024) was excluded from the severe dysplasia subgroup as outcomes were reported jointly for moderate-to-severe lesions and combined response categories [29,38].

### 3.4. Complete Response (CR)

CR rates were synthesized using a random-effects model. In the overall analysis, the pooled CR rate after photodynamic therapy was 0.34 (95% CI 0.18–0.53), with substantial heterogeneity (I^2^ = 67.1%).

When stratified by dysplasia grade, the pooled CR rate was 0.28 (95% CI 0.04–0.59; I^2^ = 71.5%) for mild dysplasia, 0.41 (95% CI 0.10–0.75; I^2^ = 0.0%) for moderate dysplasia, and 0.82 (95% CI 0.36–1.00; I^2^ = 0.0%) for severe dysplasia.

**Figure 7:**
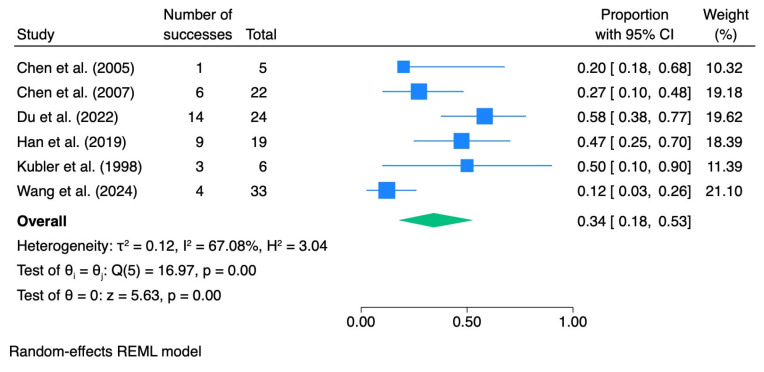
Forest plot—CR all dysplasia. Blue squares represent individual study effect estimates with 95% confidence intervals; the green diamond represents the pooled overall effect estimate with its 95% confidence interval [29,37,38,39,40,41].

**Figure 8:**
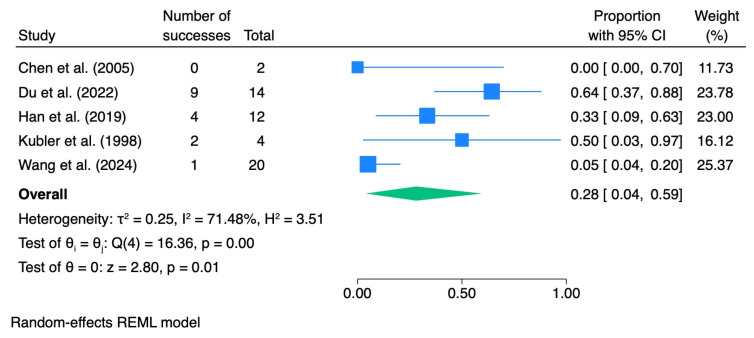
Forest plot—CR mild dysplasia. Blue squares represent individual study effect estimates with 95% confidence intervals; the green diamond represents the pooled overall effect estimate with its 95% confidence interval [29,37,39,40,41].

**Figure 9:**
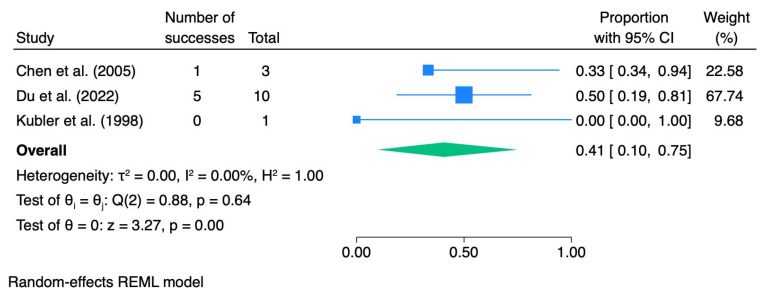
Forest plot—CR moderate dysplasia. Blue squares represent individual study effect estimates with 95% confidence intervals; the green diamond represents the pooled overall effect estimate with its 95% confidence interval [37,39,41].

**Figure 10:**
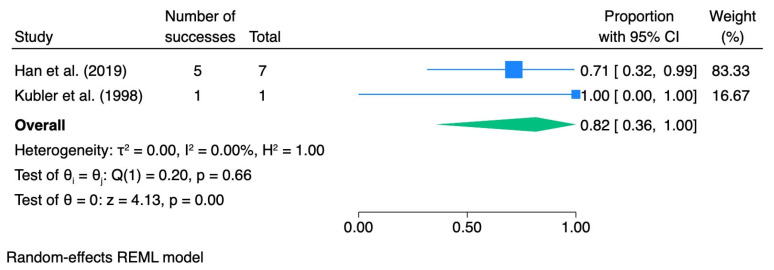
Forest plot—CR severe dysplasia. Blue squares represent individual study effect estimates with 95% confidence intervals; the green diamond represents the pooled overall effect estimate with its 95% confidence interval [40,41].

Table 5 summarizes the meta-analytic results, and the corresponding forest plots are presented in Figure 7, Figure 8, Figure 9 and Figure 10.

For subgroup-specific analyses, the same exclusions as in the ORR analysis were applied to avoid double counting and outcome misclassification.

### 3.5. Recurrence and Malignant Transformation 

Recurrence outcomes were reported in two studies (Table 6) and were therefore analyzed descriptively using incidence rates. Based on the reported mean follow-up times, recurrence incidence ranged from 0.0 per 100 person-years (Du et al., 2022) to 21.5 per 100 person-years (Wang et al., 2024) [29,39]. When pooled as a crude aggregate across these two cohorts, the recurrence incidence rate was 13.9 per 100 person-years (95% CI 7.2–24.3).

Malignant transformation was reported in one study (Wang et al., 2024; 2 events) and thus could not be meta-analyzed [29]. Assuming a mean follow-up of 20.3 months, the estimated malignant transformation incidence rate was 3.58 per 100 person-years (95% CI 0.4–12.9). In this review, we also included one study (Song et al., 2024; 54 events) focused on only malignant transformation rates; thus, it has insufficient data to be included in meta-analysis [42]. Assuming a mean follow-up of 51.7 months, the estimated malignant transformation incidence rate was 3.4 per 100 person-years (95% CI 2.6–4.4). Malignant transformation in these two studies is summarized in Table 7.

### 3.6. Sensitivity Analysis

Sensitivity analyses were performed because the initial meta-analyses for CR demonstrated substantial heterogeneity (I^2^ > 50%). A leave-one-out approach using a random-effects model with Freeman-Tukey double arcsine transformation was applied to evaluate the influence of individual studies on pooled estimates and between-study variability.

For CR across all grades of dysplasia, sequential omission of individual studies did not materially change the direction of the pooled effect. However, exclusion of the study by Wang et al. (2024) resulted in a noticeable reduction in heterogeneity (I^2^ decreased from approximately 67% to 27%) and an increase in the pooled CR estimate. Removal of other studies produced only minor fluctuations in both effect size and heterogeneity (Table 8) [29].

For CR in mild dysplasia, a similar pattern was observed. Exclusion of Wang et al. (2024) markedly reduced heterogeneity (I^2^ from approximately 71% to 15%) and increased the pooled CR proportion, whereas omission of the remaining studies led to comparatively modest changes [29]. Despite these variations, the pooled estimates across all leave-one-out scenarios remained within overlapping confidence intervals, indicating overall stability of the findings (Table 9).

### 3.7. GRADE Assessment

The GRADE assessment indicated very low certainty of evidence for the effectiveness of 5-ALA-PDT in the management of OL with histopathologically confirmed OED [43]. Although pooled estimates for ORR and CR suggested clinically meaningful treatment effects and the direction of effect was consistent across dysplasia grades, the certainty of evidence was downgraded for risk of bias, inconsistency, and imprecision.

Risk of bias was considered serious because the evidence was derived exclusively from single-arm prospective or retrospective clinical studies without randomization or control groups. Despite the use of the JBI checklist, which classified two studies as low risk, two as moderate risk, and two as high risk of bias, important methodological limitations remained, including variability in PDT protocols, follow-up duration, and outcome definitions, as well as potential selection and measurement bias inherent to uncontrolled designs.

Inconsistency was judged as serious due to the substantial heterogeneity observed in CR analyses, particularly for CR all (I^2^ = 67%) and CR mild (I^2^ = 71%). Although the direction of pooled effects consistently favored PDT, the magnitude of response varied notably between studies. Sensitivity analyses demonstrated that individual cohorts, particularly the largest study, contributed disproportionately to between-study variability. However, heterogeneity was reduced but not completely eliminated after exclusion.

Imprecision was also rated serious because several subgroup analyses were informed by a small number of studies with limited total sample sizes, resulting in wide 95% CI. Grade-specific CR estimates showed broad ranges that limited the precision of effect estimation. In addition, no clinically established decision thresholds defining adequate ORR or CR for PDT in dysplastic OL are currently available, which further constrains the interpretability and applicability of pooled proportions for clinical decision-making.

Publication bias could not be formally assessed because each meta-analysis included fewer than ten studies; therefore, its presence cannot be excluded. Overall, although pooled estimates indicated a high ORR and a moderate CR following 5-ALA PDT, the GRADE rating remained very low due to non-comparative study designs, substantial heterogeneity in key outcomes, and limited information size. Domain-level GRADE judgments are provided in Supplementary File S2.

## 4. Discussion

To our knowledge, this meta-analysis represents the first systematic and quantitative evaluation specifically dedicated to ALA-PDT in OL with histopathologically confirmed epithelial dysplasia as an explicit eligibility criterion. Previous systematic reviews have generally assessed photodynamic therapy across multiple photosensitizers and broader leukoplakia populations, and although some analyses included subgroup evaluations according to dysplasia status, dysplasia was not consistently applied as a primary inclusion filter [29,31,32]. This distinction is methodologically important, as mixing lesions with and without epithelial dysplasia increases clinical heterogeneity and may obscure treatment-specific effects in the subgroup with the highest malignant potential. Restricting eligibility to dysplasia-confirmed lesions is of major clinical relevance, as the presence of dysplasia is one of the strongest predictors of malignant transformation and largely determines both therapeutic strategy and the intensity of subsequent surveillance [43,44,45,46]. Leukoplakia with dysplasia constitutes a serious clinical problem not only because of its potential progression to squamous cell carcinoma at the site of the primary lesion, but also in the context of field cancerization, which is associated with an increased risk of tumor development in other regions of the oral cavity and upper aerodigestive tract [47,48,49,50]. Surgical excision remains the current gold standard of management. However, this approach is burdensome for patients, may result in scarring and functional or aesthetic impairment and, importantly, does not completely eliminate the risk of recurrence or malignant transformation [51,52]. These limitations justify the search for effective yet minimally invasive therapeutic modalities such as photodynamic therapy, particularly in the case of extensive, multifocal lesions or those located in anatomically challenging areas [53,54,55].

In the present analysis, a high pooled overall response rate (ORR = 0.85) was observed, indicating that ALA-PDT is frequently associated with clinical regression of dysplastic OL. Between-study heterogeneity for ORR was low to moderate (I^2^ ≈ 33%), suggesting a relatively consistent direction of effect despite variations in treatment protocols. Although numerically higher ORR values were noted in lesions with moderate and severe dysplasia, these differences should be interpreted with caution because subgroup estimates were derived from a limited number of studies and were characterized by wide confidence intervals.

In contrast, the pooled complete response (CR) rate was substantially lower (CR = 0.34) and demonstrated considerable heterogeneity (I^2^ ≈ 67%), indicating marked variability in the probability of full lesion resolution across cohorts. The highest CR proportion was observed in the severe dysplasia subgroup; however, this estimate was based on a very small evidence base and exhibited broad confidence intervals, precluding definitive conclusions regarding a true efficacy gradient according to dysplasia grade. Sensitivity analyses further indicated that between-study variability in CR was largely driven by a single influential cohort, while the overall direction of the pooled effect remained stable across leave-one-out scenarios.

It should also be emphasized that heterogeneity estimates approaching 0% in certain grade-specific subgroup analyses should not be interpreted as evidence of true homogeneity. Several of these pooled proportions were informed by only two contributing studies, a context in which statistical measures of between-study variability are inherently unstable and of limited interpretative value. Accordingly, these subgroup syntheses should be regarded as exploratory and primarily descriptive, intended to illustrate the potential direction and approximate magnitude of effect rather than to provide definitive quantitative conclusions.

Despite the variability in protocols and outcomes, the available evidence consistently indicates that PDT is an effective treatment modality for dysplastic oral leukoplakia. There are also biological mechanisms that may explain the favorable response of dysplastic epithelium to photodynamic therapy. Dysplastic epithelium may be characterized by widened intercellular spaces and a relatively thinner keratin layer, features that could facilitate deeper penetration of topically applied 5-ALA, the prodrug used in PDT [28,38,56]. After cellular uptake, exogenous ALA enters the heme biosynthesis pathway and is enzymatically converted to PpIX, the actual photoactive molecule responsible for light-induced cytotoxicity. It has been proposed that dysplastic cells, owing to altered metabolic activity and potentially reduced ferrochelatase activity, may accumulate higher intracellular levels of PpIX, which in turn could enhance photosensitivity and treatment response. However, these mechanisms are primarily derived from experimental and translational observations and should be interpreted as plausible biological explanations rather than definitive clinical determinants of therapeutic efficacy [57,58,59].

The recurrence rate after ALA-PDT, expressed as a pooled incidence of 13.9 per 100 person-years, should be interpreted with considerable caution, as only two studies provided data suitable for quantitative synthesis and the duration of follow-up varied substantially between cohorts. Differences in observation periods and surveillance intensity may meaningfully influence the estimated incidence and limit direct comparability of results.

A recent meta-analysis of OL identified female sex, older age, non-homogeneous clinical type, and incomplete surgical excision as factors associated with recurrence [60]. PDT differs from surgical management in that it does not rely on tissue resection or margin definition; instead, a photosensitizer is applied to the entire lesion surface, enabling illumination of the clinically affected field. Nevertheless, recurrences continue to be observed after PDT, which underscores the multifactorial nature of disease persistence and highlights the importance of long-term follow-up and regular clinical surveillance in treated patients.

Evidence regarding malignant transformation following ALA-PDT remains limited and is derived from a small number of observational cohorts. Therefore, available estimates should be interpreted with caution. In the study by Wang et al. (2024), the estimated incidence was 3.58 per 100 person-years with wide confidence intervals, whereas Song et al. (2024) reported an incidence of 3.4 per 100 person-years over a longer follow-up period [29,42]. Differences in study design, follow-up duration and reporting methodology preclude direct quantitative comparisons and do not allow firm conclusions regarding the effect of PDT on long-term carcinogenic risk.

In the cohort reported by Wang et al. (2024), malignant events were observed predominantly in lesions located on the floor of the mouth, an anatomical site generally regarded as higher risk, similar to the lateral and ventral tongue, retromolar trigone, and soft palate [9,61,62,63]. Additional patient- and lesion-related factors may also contribute to malignant potential—for example, prior radiotherapy has been associated with an increased risk of transformation [64]. Moreover, comparative data from surgical cohorts indicate that malignant transformation can occur despite excision, underscoring that no currently available management strategy completely eliminates carcinogenic risk [65].

Taken together, these observations support the need for regular, long-term clinical surveillance of patients treated with PDT—particularly for lesions in anatomically high-risk sites—while acknowledging the limited and non-comparative nature of the current evidence base. Attention to dynamic clinical changes (e.g., transition to non-homogeneous or verrucous morphology, development of erosions or ulcerations, or accelerated growth) and consideration of repeat biopsy when indicated remain prudent. The future integration of validated molecular biomarkers may further refine risk stratification and help tailor surveillance intensity [66,67].

5-ALA is a precursor in the heme biosynthesis pathway that is endogenously present in the human body and is enzymatically converted intracellularly to the active photosensitizer protoporphyrin IX, which contributes to a generally favorable safety profile of ALA-PDT [68,69,70,71]. The efficacy of ALA-PDT has also been demonstrated in other potentially malignant oral disorders, such as oral lichen planus [72,73,74,75,76]. At present, however, no standardized ALA-PDT protocol for oral leukoplakia exists [77].

In the included studies, treatment protocols differed substantially and were often incompletely reported. Although a 20% ALA concentration was used consistently, important technical parameters—including formulation, incubation time, fluence rate, cumulative light dose, and treatment schedule—varied or were not specified. Reported fluence rates ranged approximately from 100 to 500 mW/cm^2^, total session duration from about 300 to 1000 s, and cumulative light doses, where available, showed broad variability. The number and frequency of treatment sessions also differed markedly between cohorts. Such heterogeneity, together with incomplete reporting of formulation characteristics, limits direct comparisons and may partly explain the variability observed in complete response rates.

An important methodological aspect influencing the practical feasibility of ALA-PDT in the oral cavity is the formulation of the photosensitizer and its retention at the lesion site. In the present review, only three included studies explicitly reported both the formulation and incubation time of ALA, namely gel (Han et al., 2019; Wang et al., 2024) and cream (Kübler et al., 1998), each applying an approximately 2 h incubation period [29,40,41]. The limited availability of such detailed reporting highlights a substantial gap in the literature and restricts direct comparisons regarding formulation-dependent tolerability.

A frequently described feature across studies was a relatively long incubation period of approximately 1.5–2 h. In the oral environment, the practical tolerability of this interval is closely linked to the delivery vehicle and local intraoral conditions rather than exposure time alone. Continuous salivary flow, mucosal movement, and epithelial desquamation may reduce topical drug retention, particularly when formulations lack sufficient mucoadhesive properties. Broader technical and narrative literature indicates that the effectiveness of topical ALA administration in the oral cavity is likely influenced by salivary dilution, mucosal movement, and insufficient adhesion of commercially available products [27,54]. Gel-based preparations are most frequently described, probably due to their partial resistance to salivary clearance and improved mucosal contact compared with aqueous solutions or mouth rinses, which are more commonly employed for diagnostic fluorescence rather than therapeutic purposes [75,78,79,80]. Importantly, even gel formulations often require auxiliary measures such as cotton roll isolation, suction control, or protective gauze to maintain adequate drug localization, indicating that formulation alone does not fully eliminate retention challenges [80]. Consequently, logistical challenges associated with incubation should be interpreted as an interaction between formulation characteristics, incubation duration, and local intraoral conditions rather than as an inherent limitation of ALA-PDT itself.

Various mucoadhesive and polymer-based delivery platforms, including emulgel formulations, are currently being developed to enhance photosensitizer persistence at the lesion site and improve patient comfort by potentially shortening the required incubation time [81]. Emerging approaches of this type have been described in the broader pharmaceutical and biomaterials literature. Preliminary clinical observations in other oral mucosal disorders, such as oral lichen planus, have reported encouraging outcomes with such strategies [82,83]. Nevertheless, these methods remain underrepresented in controlled clinical trials and require further systematic evaluation before firm conclusions regarding their comparative effectiveness can be drawn.

An additional advantage of topical ALA-PDT is the generally low frequency and mild nature of adverse effects, such as a transient burning sensation, short-term photosensitivity, or superficial epithelial desquamation, which are typically self-limiting [84,85,86,87]. The procedure can be safely repeated, and in cases of insufficient response, surgical excision remains a viable subsequent option.

Tobacco smoking is a major modifying factor for the development and progression of oral potentially malignant disorders [86,87,88,89,90]. Although smoking status was incompletely reported in the included studies, available data indicate that smoking does not significantly affect the clinical response to ALA-PDT in oral leukoplakia [24,27,28,35,86,91]. Nevertheless, tobacco use remains one of the principal drivers of malignant transformation; therefore, smoking status should always be considered in risk assessment and long-term follow-up planning, and patients should be routinely provided with smoking-cessation counseling at each visit.

### 4.1. Limitations

The main limitations of this meta-analysis stem from the restricted size and structure of the available evidence base. Only six eligible studies comprising 109 patients were identified, which limits the statistical power and precision of pooled estimates. Furthermore, the evidence was derived exclusively from single-arm observational cohorts without control groups, precluding direct comparative conclusions regarding the relative effectiveness of ALA-PDT versus alternative management strategies. This design structure also introduces an inherent risk of publication bias, as small single-arm studies reporting favorable outcomes are more likely to be published than those demonstrating limited or absent clinical response. Consequently, the pooled estimates—particularly the high overall response rate—should be interpreted with caution, as they may overrepresent the more favorable end of the therapeutic spectrum.

Additional limitations include substantial heterogeneity of treatment protocols and incomplete reporting of key methodological and clinical parameters, including formulation characteristics, lesion-level data, and modifying factors such as smoking status. These inconsistencies limited the ability to perform formal meta-regression analyses and restricted the identification of reliable predictors of treatment response.

Recurrence and malignant transformation outcomes were based on a very small number of studies and were therefore analyzed descriptively rather than through formal meta-analytic pooling. Variable follow-up durations further reduce the comparability and precision of these incidence estimates. The overall certainty of evidence, as assessed by GRADE, was rated as very low, primarily due to non-comparative study designs, heterogeneity, and limited information size.

Finally, although a comprehensive search strategy was applied, Embase and gray literature sources were not systematically analyzed, which may have contributed to the omission of unpublished or non-indexed studies and further increased the risk of publication bias. Taken together, these factors indicate that the pooled proportions presented in this review should be interpreted as descriptive indicators of treatment response rather than definitive measures of clinical effectiveness.

### 4.2. Future Directions

Future research should focus on large, multicenter studies with standardized and comprehensive methodological reporting, enabling the development of a uniform ALA-PDT protocol that considers irradiation parameters, number of sessions, incubation time and lesion characteristics. Systematic collection of data on risk factors, including tobacco use, is also essential.

## 5. Conclusions

In conclusion, although the certainty of the available evidence remains low and the present findings should be interpreted with caution, photodynamic therapy with ALA-PDT appears to be a clinically meaningful and minimally invasive treatment option for oral leukoplakia with epithelial dysplasia. The pooled results suggest a high likelihood of clinical response, accompanied by a favorable safety profile and the possibility of repeated application without compromising subsequent surgical management.

Surgical excision remains the current gold standard, particularly for lesions with high oncological risk, rapid progression or lack of response to conservative measures. Nevertheless, ALA-PDT may be reasonably considered in selected patients—especially in cases of low-grade dysplasia, multifocal disease, or lesions located in anatomically or functionally sensitive areas—where a minimally invasive approach may offer a balanced alternative between therapeutic intervention and preservation of oral structures. Importantly, the use of PDT does not preclude later surgical treatment if required.

Regardless of the chosen management strategy, all patients with epithelial dysplasia require careful and long-term clinical surveillance. Future well-designed controlled trials and standardized treatment protocols are necessary to more precisely define the role of ALA-PDT within the therapeutic algorithm for dysplastic oral leukoplakia.

## Figures and Tables

**Table 1:** Inclusion and exclusion criteria shown using the PICO framework.

PICOS Element	Inclusion Criteria	Exclusion Criteria
P-Population	Adults (≥18 years) Clinical oral leukoplakia Histopathologically confirmed epithelial dysplasia Lesions located in the oral mucosa	Non-dysplastic leukoplakia Other OPMDs without separate data for dysplastic OL Extraoral leukoplakia Mixed cohorts where dysplastic OL data cannot be isolated
I-Intervention	PDT as primary treatment for dysplastic OL 5-ALA as photosensitizer	PDT only as adjunct to another treatment with non-separable PDT outcomes Combined protocols where the specific effect of PDT cannot be distinguished
C-Comparison	Single-arm PDT studies Studies with comparator arms if data for the PDT arm are extractable separately	Studies including only non-PDT treatments Comparative studies where outcomes for the PDT arm are not reported separately
O-Outcomes	Clinical response: complete, partial, no response Recurrence after initial response Malignant transformation during follow-up	Studies reporting only histological or biomarker changes without any clinical outcome Outcomes unrelated to treatment effectiveness Insufficient numerical data for extraction/meta-analysis
S-Study design	Prospective or retrospective clinical studies Interventional or observational single-arm designs Case series with PDT-treated dysplastic OL lesions or patients Full-text articles in English, human subjects only	Case reports Reviews, conference abstracts Animal, in vitro or ex vivo studies Non-English publications

**Table 2:** Main characteristics of the single-arm studies included in the review, presenting demographic data, dysplasia grade and outcomes.

							Outcomes				
Study ID	Country	Mean Age ± SD (years)	Sex (M/F)	Dysplasia Grade	Mean Follow-Up (Months)	Unit of Analysis (Patient/Lesion)	Outcome Type	Mild Dysplasia	Moderate Dysplasia	Severe Dysplasia	All Dysplasia
Chen et al. (2005) [37]	Taiwan	50 ± 12	5/0	2 mild dysplasia 3 moderate dysplasia 0 severe dysplasia	10.3	patient	CR	0	1	0	1
							OR	2	3	0	5
							Rec	2	2	0	4
							M	ND	ND	ND	ND
Chen et al. (2007) [38]	Taiwan	51 ± 12	ND	13 mild dysplasia 9 moderate dysplasia 0 severe dysplasia	24.3	patient	CR	6		ND	6
							OR	20		ND	20
							Rec	ND	ND	ND	ND
							M	ND	ND	ND	ND
Du et al. (2022) [39]	China	39.32 ± 10.65	ND	14 mild dysplasia 10 moderate dysplasia 0 severe dysplasia	15.2	patient	CR	9	5	0	14
							OR	13	8	0	21
							Rec	0	0	0	0
							M	ND	ND	ND	ND
Han et al. (2019) [40]	China	56.07 ± 11.01	ND	12 mild dysplasia 0 moderate dysplasia 7 severe dysplasia	3	patient	CR	4	ND	5	9
							OR	10	ND	6	16
							Rec	ND	ND	ND	ND
							M	ND	ND	ND	ND
Kubler et al. (1998) [41]	Germany	-	6/0	4 mild dysplasia 1 moderate dysplasia 1 severe dysplasia	9	patient	CR	2	0	1	3
							OR	3	1	1	5
							Rec	ND	ND	ND	ND
							M	ND	ND	ND	ND
Wang et al. (2024) [29]	China	55.5 ± 12.7	ND	20 mild dysplasia 13 moderate to severe dysplasia	20.3	patient	CR	1	3		4
							OR	13	9		22
							Rec	6	6		12
							M	0	2		2

CR—complete response; OR—overall response; Rec—recurrence; M—malignant transformation; ND—no data. Note: In the study by Chen et al. (2007), data for mild and moderate dysplasia were reported in a combined format. In the study by Wang et al. (2024), data were reported jointly for moderate and severe dysplasia [29,38].

**Table 3:** Protocol of ALA-PDT, including DLI, 5-ALA formulation and concentration, and PDT duration and method.

Study ID	Country	Drug-Light Interval [Hours]	ALA Formulation	ALA Concentration [%]	Irradiation Cycles	Total Session Time [sec]	Fluence Rate [mW/cm^2^]	Light Exposure Dose [J/cm^2^]	Cumulative Light Dose [J/cm^2^]	Treatment Schedule
Chen et al. (2005) [37]	Taiwan	1.5	ND	20	5 × 3 min + 1 × 100 s (with 5 × 3 min rests)	1000	100	100	200–500	2–5 sessions twice a week
Chen et al. (2007) [38]	Taiwan	1.5–2	ND	20	5 × 3 min + 1 × 100 s (with 5 × 3 min rests)	1000	100	100	100–600	1–6 sessions once or twice a week
Du et al. (2022) [39]	China	ND	ND	20	ND	ND	300	100	300–500	3–5 sessions every two weeks
Han et al. (2019) [40]	China	2	gel	20	3 min (with 1 min rests)	ND	500	90–180	90–540	1–3 sessions Every two weeks
Kubler et al. (1998) [41]	Germany	2	cream	20	ND	1000	100	100	100	One session (total)
Wang et al. (2024) [29]	China	2	gel	20	ND	300	150–300	ND	ND	3–4 sessions every one or two weeks

ND—no data; DLI—drug-light interval.

**Table 4:** Overall response rate (ORR) after photodynamic therapy for dysplastic oral leukoplakia.

		95% CI			
	*p*	LL	UL	H^2^	I^2^ (%)
All dysplasia	0.85	0.74	0.93	1.50	33.40
Mild dysplasia	0.83	0.67	0.96	1.30	23.02
Moderate dysplasia	0.93	0.64	1.00	1.00	0.00
Severe dysplasia	0.95	0.54	1.00	1.00	0.00

**Table 5:** Complete response (CR) after photodynamic therapy for dysplastic oral leukoplakia.

		95% CI			
	*p*	LL	UL	H^2^	I^2^(%)
All dysplasia	0.34	0.18	0.53	3.04	67.08
Mild dysplasia	0.28	0.04	0.59	3.51	71.48
Moderate dysplasia	0.41	0.1	0.75	1.00	0.00
Severe dysplasia	0.82	0.36	1.00	1.00	0.00

**Table 6:** Incidence rate of recurrence after photosdynamic therapy for oral leukoplakia.

Study	Recurrences (E)	N	Follow-Up (Months)	Person-Years	Incidence Rate/100 PY (95% CI)
Du et al. (2022) [39]	0	24	15.2	30.40	0.0 (0.0–12.1)
Wang et al. (2024) [29]	12	33	20.3	55.83	21.5 (11.1–37.5)
Overall	12	57	-	86.23	13.9 (7.2–24.3)

Incidence rates (IRs) were calculated as the number of recurrences divided by the estimated person-time (N × mean follow-up in years) and expressed per 100 person-years with 95% Poisson confidence intervals.

**Table 7:** Malignant transformation after photodynamic therapy for oral leukoplakia.

Study	Malignant Transformation (E)	N	Follow-Up (Months)	Person-Years	Incidence Rate/100 PY (95% CI)
Wang et al. (2024) [29]	2	33	20.3	55.77	3.58 (0.4–12.9)
Song et al. (2024) [42]	54	367	51.7	1581.77	3.4 (2.6–4.4)

Incidence rates (IRs) were calculated as the number of recurrences divided by the estimated person-time (N × mean follow-up in years) and expressed per 100 person-years with 95% Poisson confidence intervals.

**Table 8:** Leave-one-out sensitivity analysis for CR—all studies.

Omitted Study	New Pooled CR (95% CI)	New Heterogeneity (I^2^, %)
Chen et al. (2005) [37]	0.37 (0.19–0.57)	76.1
Chen et al. (2007) [38]	0.38 (0.18–0.60)	75.8
Du et al. (2022) [39]	0.30 (0.16–0.47)	56.3
Han et al. (2019) [40]	0.33 (0.15–0.54)	73.3
Kübler et al. (1998) [41]	0.34 (0.17–0.54)	75.2
Wang et al. (2024) [29]	0.43 (0.30–0.57)	26.7

**Table 9:** Leave-one-out sensitivity analysis for CR—mild dysplasia.

Omitted Study	New Pooled CR (95% CI)	New Heterogeneity (I^2^, %)
Chen et al. (2005) [37]	0.35 (0.09–0.67)	80.6
Du et al. (2022) [39]	0.23 (0.06–0.47)	52.2
Han et al. (2019) [40]	0.32 (0.04–0.70)	80.7
Kübler et al. (1998) [41]	0.29 (0.06–0.62)	79.8
Wang et al. (2024) [29]	0.47 (0.29–0.65)	14.6

## Data Availability

Data are contained within the Systematic Review.

## Supplementary Materials

The following supporting information can be downloaded at https://www.mdpi.com/article/10.3390/pharmaceutics18020254/s1, File S1: PRISMA checklist [34]; File S2: GRADE assessment [43].
