# Supplementary material for: Efficacy of 5-ALA Photodynamic Therapy in Dysplastic Oral Leukoplakia: Systematic Review and Meta-Analysis
Source: Pharmaceutics. 2026 Feb 18;18(2):254. doi: 10.3390/pharmaceutics18020254 (PMC12944706; doi:10.3390/pharmaceutics18020254)
Supplement: Supplementary file 1 [file pharmaceutics-18-00254-s001.zip › pharmaceutics-4147153 - GRADE assessment.pdf]

Supplementary File S2 – GRADE Evidence Profile

This table summarizes the GRADE assessment for the overall body of evidence regarding the effectiveness of ALA-PDT in the management of oral leukoplakia with histopathologically confirmed epithelial dysplasia. The GRADE approach was applied to the evidence as a whole rather than to individual outcomes. Judgments were based on study design, Joanna Briggs Institute (JBI) risk-of-bias assessment, pooled proportions for overall response rate (ORR) and complete response (CR), heterogeneity statistics (I^2), confidence intervals and consistency of findings across dysplasia grades. Only studies included in the quantitative synthesis were considered.

GRADE Domain	Judgment	Rationale	Impact on Certainty
Risk of Bias	Serious	All included studies were single-arm prospective or retrospective clinical series without randomization or control groups. Two studies were low risk, two moderate, and two high risk according to the JBI checklist. Variability in treatment protocols, follow-up duration, and outcome definitions may have introduced bias.	Downgraded one level
Inconsistency	Serious	Substantial heterogeneity was observed for complete response outcomes ($I^2 \approx 67\%$ overall and ≈71% for mild dysplasia). The direction of effect	Downgraded one level

		consistently favored PDT, but magnitude varied notably across studies. Sensitivity analyses reduced but did not eliminate heterogeneity.	
Indirectness	Not serious	All studies directly evaluated topical ALA-PDT in patients with histopathologically confirmed oral epithelial dysplasia and reported clinically relevant outcomes, closely matching the review question.	No downgrade
Imprecision	Serious	Several subgroup analyses were based on a small number of studies and limited total sample sizes, resulting in wide confidence intervals, particularly in grade-specific CR analyses.	Downgraded one level
Publication Bias	Undetected	Formal assessment of publication bias was not feasible due to the small number of studies per outcome (k < 10). Publication bias cannot be excluded.	No downgrade

Overall Certainty of Evidence: VERY LOW
